# Breath biopsy of breast cancer using sensor array signals and machine learning analysis

**DOI:** 10.1038/s41598-020-80570-0

**Published:** 2021-01-08

**Authors:** Hsiao-Yu Yang, Yi-Chia Wang, Hsin-Yi Peng, Chi-Hsiang Huang

**Affiliations:** 1 Institute of Environmental and Occupational Health Sciences, National Taiwan University College of Public Health, Taipei, Taiwan; 2 Department of Environmental and Occupational Medicine, National Taiwan University Hospital, Taipei, Taiwan; 3 Department of Anesthesiology, National Taiwan University College of Medicine, Taipei, Taiwan; 4 Department of Anesthesiology, National Taiwan University Hospital, Taipei, Taiwan

**Keywords:** Biological techniques, Biotechnology, Cancer, Health care

## Abstract

Breast cancer causes metabolic alteration, and volatile metabolites in the breath of patients may be used to diagnose breast cancer. The objective of this study was to develop a new breath test for breast cancer by analyzing volatile metabolites in the exhaled breath. We collected alveolar air from breast cancer patients and non-cancer controls and analyzed the volatile metabolites with an electronic nose composed of 32 carbon nanotubes sensors. We used machine learning techniques to build prediction models for breast cancer and its molecular phenotyping. Between July 2016 and June 2018, we enrolled a total of 899 subjects. Using the random forest model, the prediction accuracy of breast cancer in the test set was 91% (95% CI: 0.85–0.95), sensitivity was 86%, specificity was 97%, positive predictive value was 97%, negative predictive value was 97%, the area under the receiver operating curve was 0.99 (95% CI: 0.99–1.00), and the kappa value was 0.83. The leave-one-out cross-validated discrimination accuracy and reliability of molecular phenotyping of breast cancer were 88.5 ± 12.1% and 0.77 ± 0.23, respectively. Breath tests with electronic noses can be applied intraoperatively to discriminate breast cancer and molecular subtype and support the medical staff to choose the best therapeutic decision.

## Introduction

Breast cancer is the most commonly diagnosed cancer and the leading cause of cancer death among females^1^. Early detection can improve treatment and decrease mortality^2^. The molecular subtype is an independent prognostic factor of breast cancer^3,4^. Detecting the expression of estrogen receptor (ER) and progesterone receptor (PR), and overexpression of human epidermal growth factor receptor 2 (HER2) has been used to guide the therapy decisions^5,6^. Based on the expression of receptors, breast cancer can be further classified into distinct molecular subtypes, which include luminal A, luminal B, HER2, and triple-negative^7^. Metabolic alterations are observed in different molecular subtypes and histological types of breast cancer^8^. Fan et al. analyzed the metabolites in plasma of breast cancer and identified eight metabolites for the classification of breast cancer subtypes^9^. An in vitro study showed that breast cancer cells of different statuses could generate specific volatile metabolites^10^.

**Figure 1:**
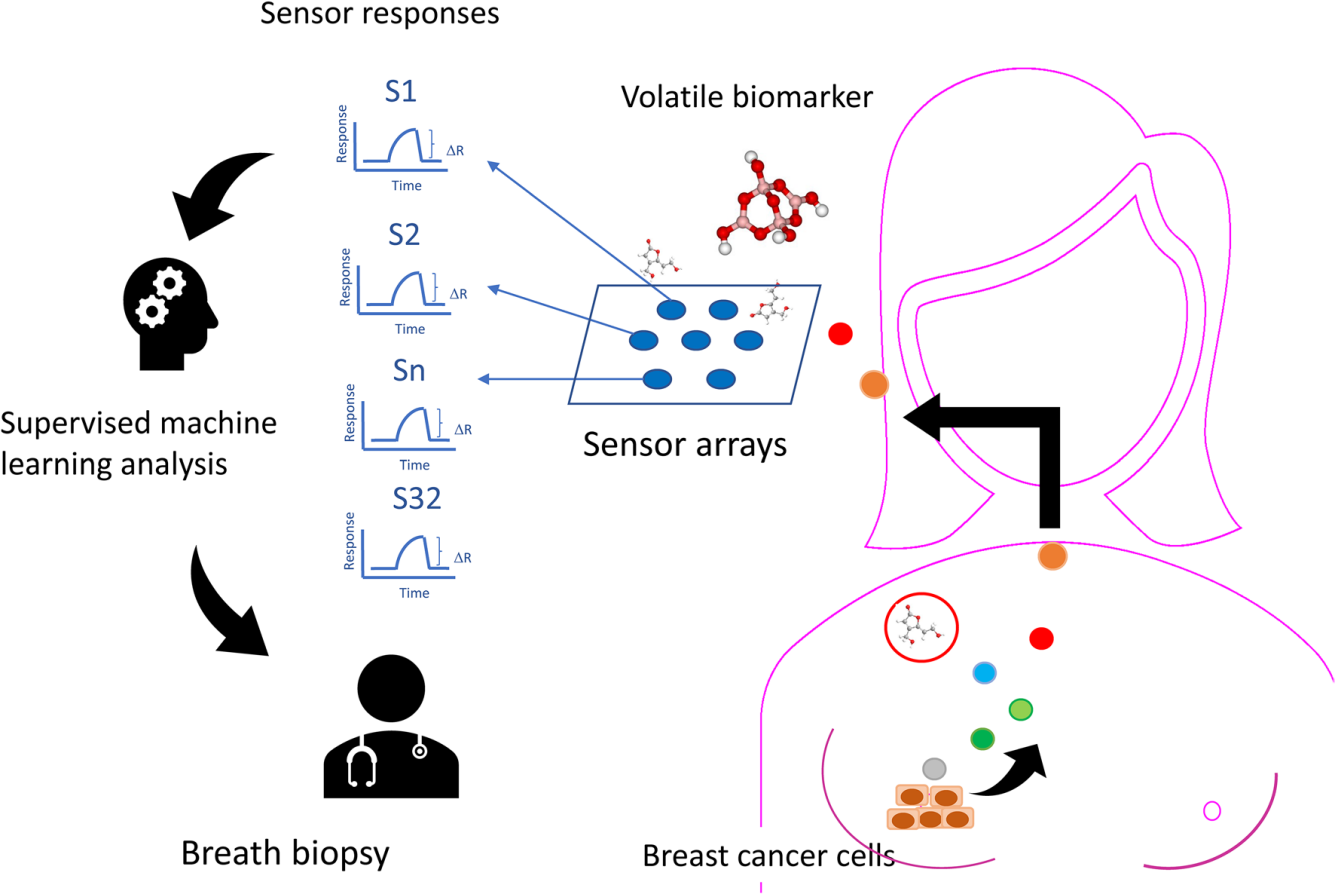
Graphical abstract showing the principle of breath biopsy. Legends: Volatile metabolites produced by breast cancer cells circulate to the lungs and are released into the breath. Using the sensor array to detect the pattern of exhaled volatile biomarkers, we can detect the molecular type of breast cancer early by collecting alveolar air during surgery.

Breathomics is an emerging science to diagnose diseases by analyzing volatile metabolites produced by changes in metabolic processes caused by disease^11^. The volatile metabolites produced during the physiological and pathological processes of the lung diseases are released into the alveolar air^12^. The volatile metabolites produced by tumors have the potential to serve as noninvasive biomarkers^11^. The gas chromatography-mass spectrometry (GC–MS) and electronic nose (E-nose) are two methods to analyze these volatile metabolites. The electronic nose uses a fingerprinting approach to explore the exhaled breath by sensor arrays. When the volatile metabolites from a breath sample are presented to the E-nose sensor array, the chemicals interact with the sensors and change their electric resistance. The data are processed by machine learning techniques to predict the probability of the diagnosis of a disease^13^. Due to non-invasiveness and rapid diagnosis, there is increasing interest in the analysis of volatile metabolites in exhaled breath to diagnose diseases^14^. The objective of this study was to develop a breath test to detect breast cancer and its molecular subtype. We analyzed the patient’s alveolar air through an electronic nose and applied machine learning statistics to build a predictive model for the diagnosis of breast cancer (Fig. 1).

## Results

**Figure 2:**
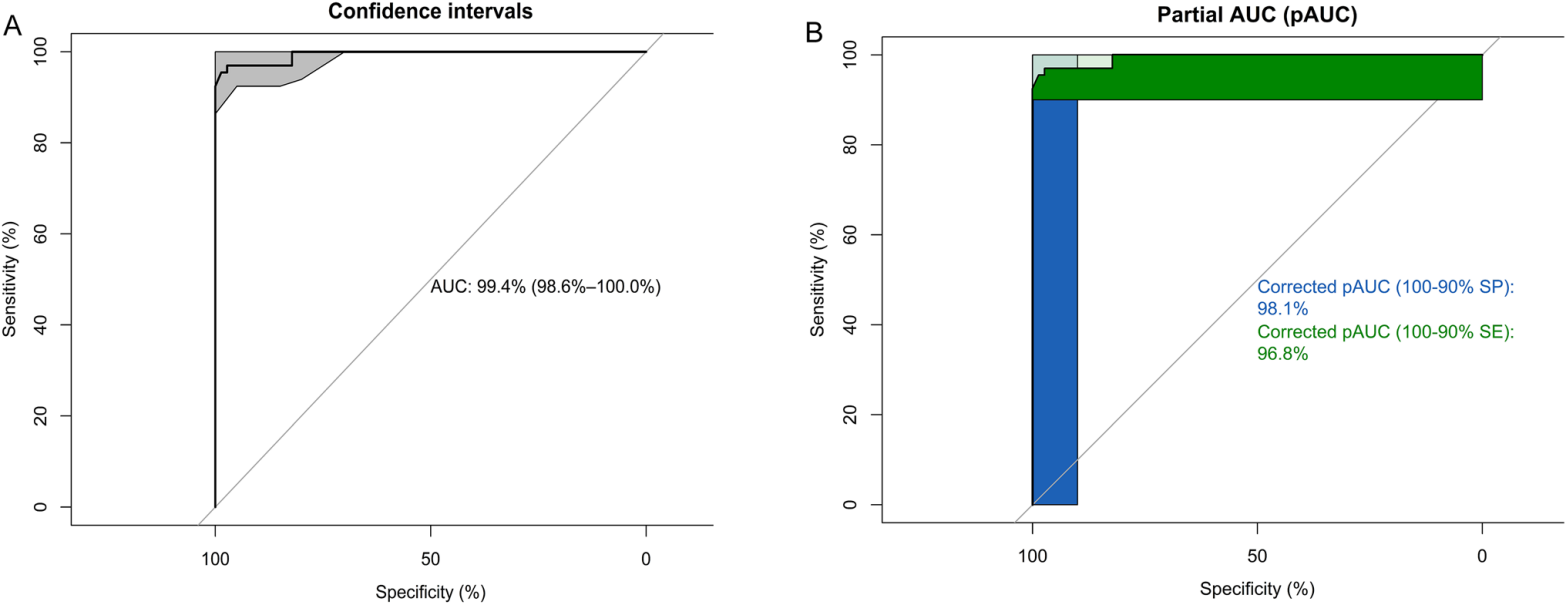
Statistical model performance of the random forest algorithm to diagnose breast cancer. Legends: (**A**) The discriminatory accuracy is expressed as AUC with the 95% confidence interval. The grey area is the 95% confidence intervals using bootstrap resampling for 2000 replicates. (**B**) The partial area under the receiver operating curve (pAUC). The blue area corresponds to the pAUC region between 90 and 100% for specificity (SP), and the green area corresponds to the pAUC region between 90 and 100% for sensitivity (SE). The corrected pAUCs are printed in the middle of the plot.

Between July 2016 and June 2018, a total of 899 subjects were screened and assessed. Based on the defined inclusion and exclusion criteria, we eliminated six study subjects who did not have sensor data for technical reasons, 122 male subjects, 222 benign breast tumors, 40 subjects who had received chemotherapy, 57 current smokers, 19 former smokers, 23 second-hand smokers, 63 subjects with diabetes mellitus, and ten subjects with asthma, a total of 439 study subjects were used in the final analyses that included 351 cases of malignant breast tumor and 88 controls. The mean age of study subjects was 55.03 (SD 12.08) years. There were no statistically significant differences in age, renal and liver functions, and inflammatory status between the case group and the control group (Table 1). Using a random forest model, the prediction accuracy of breast cancer in the test set was 91%, sensitivity was 86%, specificity was 97%, positive predictive value (PPV) was 97%, negative predictive value (NPV) was 97%, and the area under the receiver operator characteristic curve (AUC) was 0.99 (95% CI: 0.97–1.00). The reliability of prediction as measured by the kappa value was 0.83 (Table 2). The 95% confidence interval of receiver operating characteristic (ROC) using bootstrap resampling for 2000 replicates was shown in Fig. 2. The partial area under the receiver operating curve (pAUC) between 90 and 100% for specificity was 98.1%, and the pAUC between 90 and 100% for sensitivity was 96.8%. In the identification of molecular subtypes of breast cancer, the random forest model had the highest accuracy. The mean value of leave-one-out cross-validation accuracy was 88.5 ± 12.1%, and the kappa reliability was 0.77 ± 0.23 (Table 3).

**Table 1:** Demographic characteristics of the study subjects.

Characteristics	Case group (*n* = 351)	Control group (*n* = 88)	p value
Age (year), mean (SD)	55.35 (11.58)	55.69 (13.96)	0.31
White blood cell (10^3^/µL), mean (SD)	6.19 (1.80)	6.52 (1.67)	0.12
Blood urea nitrogen (mg/dL), mean (SD)	13.51 (5.71)	14.39 (3.91)	0.09
Creatinine (mg/dL), mean (SD)	0.67 (0.14)	0.78 (0.56)	0.07
Alanine aminotransferase (U/L), mean (SD)	18.74 (17.61)	17.00 (9.99)	0.23
Fasting sugar	100.4 (54.69)	93.88 (16.02)	0.07
Cholesterol (mg/dL)	182.6(57.90)	201.4(18.89)	0.12
**Pathology**			
Invasive carcinoma (%)	249 (63.55)	N/A	
Mucinous carcinoma (%)	5 (1.14)	N/A	
Metaplastic carcinoma (%)	2 (0.46)	N/A	
Paget disease (%)	2 (0.46)	N/A	
Ductal carcinoma in situ (DCIS) (%)	41 (9.34)	N/A	
Non-comedo DCIS (%)	1 (0.23)	N/A	
DCIS with microinvasion (%)	10 (2.28)	N/A	
Lobular Carcinoma in Situ (%)	5 (1.14)	N/A	
**Molecular subtypes**			
Luminal A (%)	106 (44.92)	N/A	
Luminal B (%)	81 (34.32)	N/A	
HER2/neu (%)	33 (13.98)	N/A	
Triple-Negative (%)	16 (6.78)	N/A

**Table 2:** Prediction accuracy of the electronic nose in the test set of machine learning algorithms.

Model and parameters	Accuracy (95% CI)	Sensitivity	Specificity	PPV	NPV	Kappa	AUC (95% CI)
*k*-nearest neighbors (*k* = 5)	0.66 (0.58–0.74)	0.48	0.86	0.80	0.60	0.34	0.78 (0.71–0.86)
Naive Bayes (fL = 0, usekernel = TRUE, adjust = 1)	0.66 (0.58–0.74)	0.79	0.52	0.64	0.69	0.31	0.78 (0.71–0.85)
Decision tree (trials = 20, model = tree, window = FALSE)	0.91 (0.85–0.95)	0.86	0.97	0.97	0.86	0.82	0.98 (0.76–1.00)
Neural network (size = 1, decay = 1e−04)	0.67 (0.61–0.77)	0.71	0.62	0.68	0.66	0.33	0.98 (0. 96–1.00)
Support vector machines (linear kernel) (C = 1)	0.65 (0.51–0.68)	0.78	0.52	0.64	0.68	0.29	0.98 (0.96–1.00)
Support vector machines (radial kernel) (sigma = 0.1040273, C = 1)	0.68 (0.59–0.75)	0.60	0.76	0.73	0.63	0.36	0.98 (0.96–1.00)
Support vector machines (polynomial kernel) (degree = 3, scale = 0.1, C = 1)	0.65 (0.60–0.73)	0.78	0.52	0.64	0.68	0.30	0.98 (0. 96–1.00)
Random forest (mtry = 2)	0.91 (0.85–0.95)	0.86	0.97	0.97	0.97	0.83	0.99 (0.99–1.00)
Mean value (SD)	0.72 (0.12)	0.73 (0.13)	0.72 (0.20)	0.76 (0.14)	0.72 (0.13)	0.45 (0.23)	0.93 (0.09)

*PPV* positive predictive value,* NPV* negative predictive value,* AUC* area under the receiver operating curve.

**Table 3:** Leave-one-out cross-validated discrimination accuracy and reliability of molecular phenotyping of breast cancer using machine learning algorithms.

Model	Luminal A		Luminal B		HER2/neu		Triple-negative	
	Accuracy	Kappa	Accuracy	Kappa	Accuracy	Kappa	Accuracy	Kappa
*k*-nearest neighbors	0.61	0.22	0.60	0.20	0.67	0.34	0.84	0.69
Naive Bayes	0.59	0.18	0.53	0.06	0.64	0.28	0.68	0.36
Decision tree	0.62	0.27	0.71	0.41	0.97	0.94	0.99	0.98
Neural network	0.55	0.11	0.56	0.12	0.65	0.29	0.77	0.54
Support vector machines (linear kernel)	0.51	0.02	0.54	0.08	0.60	0.20	0.77	0.54
Support vector machines (radial kernel)	0.57	0.15	0.59	0.18	0.59	0.19	0.84	0.67
Support vector machines (polynomial kernel)	0.63	0.26	0.63	0.25	0.66	0.32	0.85	0.69
Random forest	0.74	0.49	0.83	0.66	0.98	0.95	0.99	0.97

**Figure 3:**
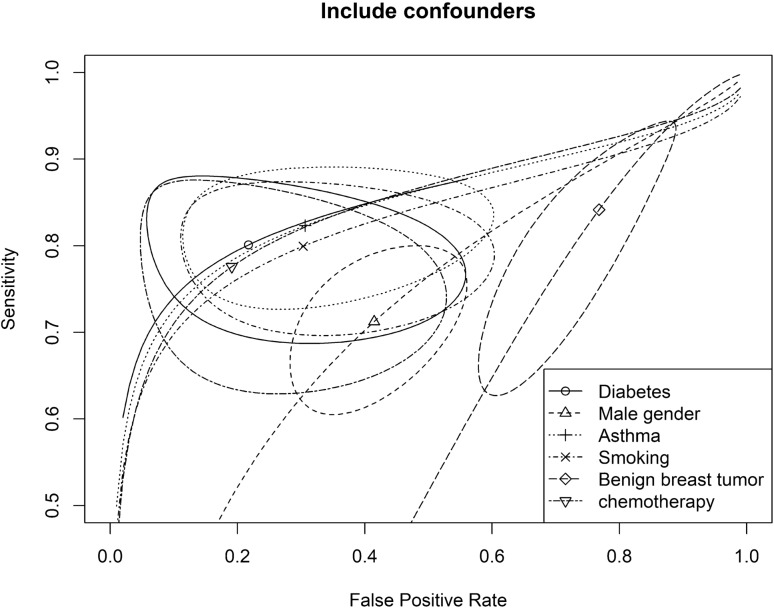
Summary receiver operating characteristic (SROC) cures for diagnostic accuracy that includes confounding factors or comorbidities. Legends: This figure shows a joint estimate of false positive rate and sensitivity for the electronic nose data with 95% confidence and prediction regions. Scatter points are the accuracy obtained from different machine learning models, and the solid closed curve is the 95% confidence region.

**Figure 4:**
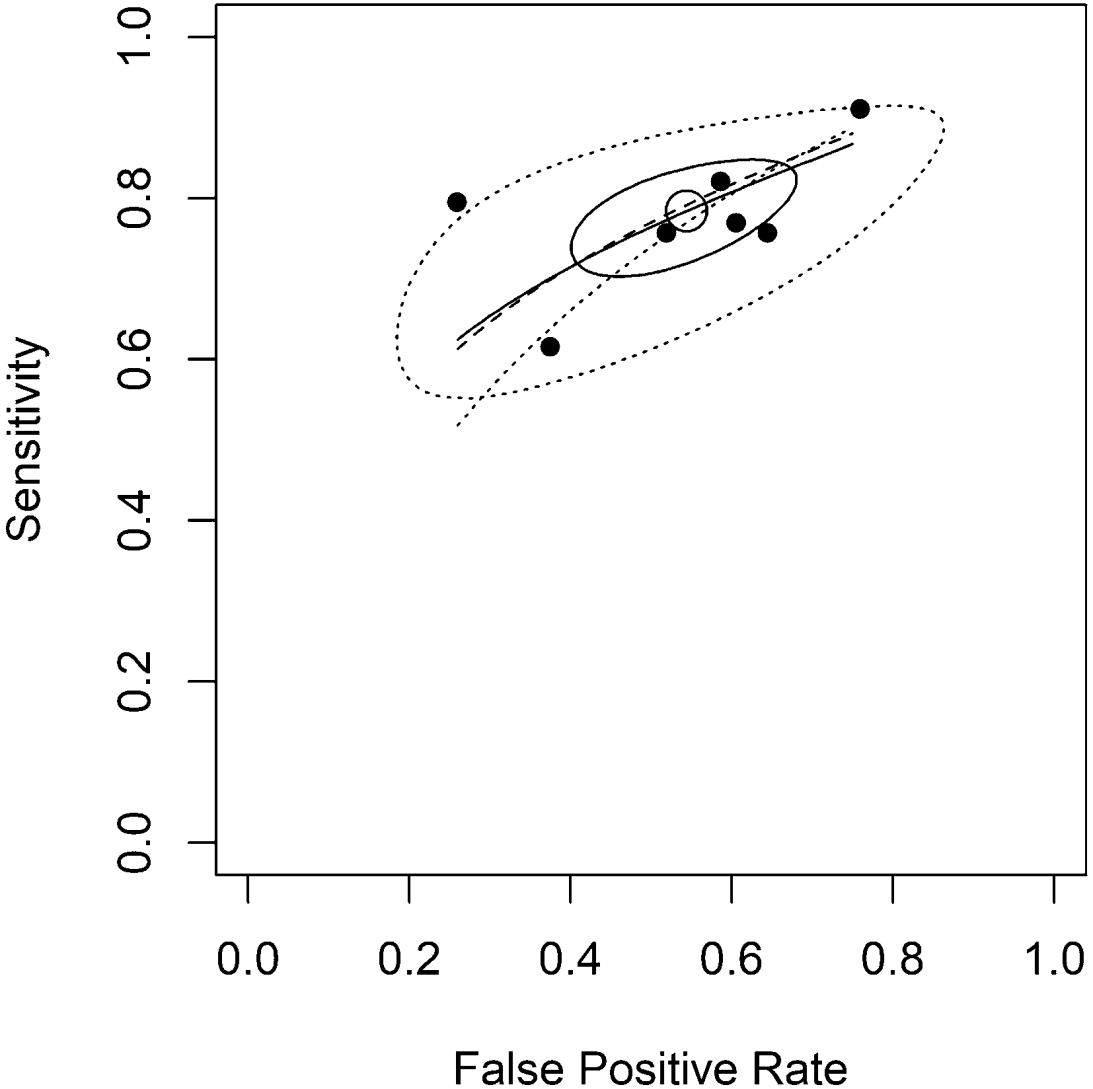
The joint estimate of false positive rate and sensitivity for the electronic nose data with 95% confidence and prediction regions. Legends: This figure shows the data that includes all study population without excluding any confounding factor or comorbidity. Scatter points are the data. A solid closed curve is the 95% confidence region. The dotted closed curve is the 95% prediction region. Three summary ROC curves are seen. The short solid line is the curve proposed by Rutter and Gatsonis^15^. The dashed line is the curve proposed by Moses et al.^16^; the dotted line is the curve proposed by Rücker and Schumacher^17^.

To evaluate the influence of comorbidities and confounding factors on diagnostic accuracy, we have used all the population and conducted additional analyses to compare the effects of comorbidities and confounding factors on diagnostic accuracy. The results showed that the inclusion of study subjects with a history of asthma did not significantly affect diagnostic accuracy. The inclusion of subjects with a history of smoking, chemotherapy, or diabetes had a moderate impact on accuracy. The inclusion of male gender and benign breast tumor significantly influenced the accuracy (Fig. 3). When we included study subjects with a history of asthma (n = 10), the diagnostic odds ratio (DOR) was 10.62. When we included study subjects with a history of smoking (n = 99), the DOR was 9.12. When we included study subjects with a history of chemotherapy (n = 40), the DOR was 8.62. When we included study subjects with diabetes (n = 63), the DOR was 8.51. When we included the male gender (n = 122), the DOR was 3.48. When we included benign breast tumors (n = 222), the DOR was 1.39. When we included all study population without excluding any comorbidity or confounding factor, the AUC was 0.72 (95% CI: 0.71–0.76). We provided the summary receiver operating characteristic (SROC) curve to show the joint estimate of the false positive rate and sensitivity for the electronic nose (Fig. 4).

## Discussion

To the best of our knowledge, this is the first study to provide evidence that the breath test can predict breast cancer and its molecular subtype with good accuracy and reliability. The breath test uses the latest breathomics and artificial intelligence (AI) technologies to assist physicians in making treatment decisions during surgery.

**Figure 5:**
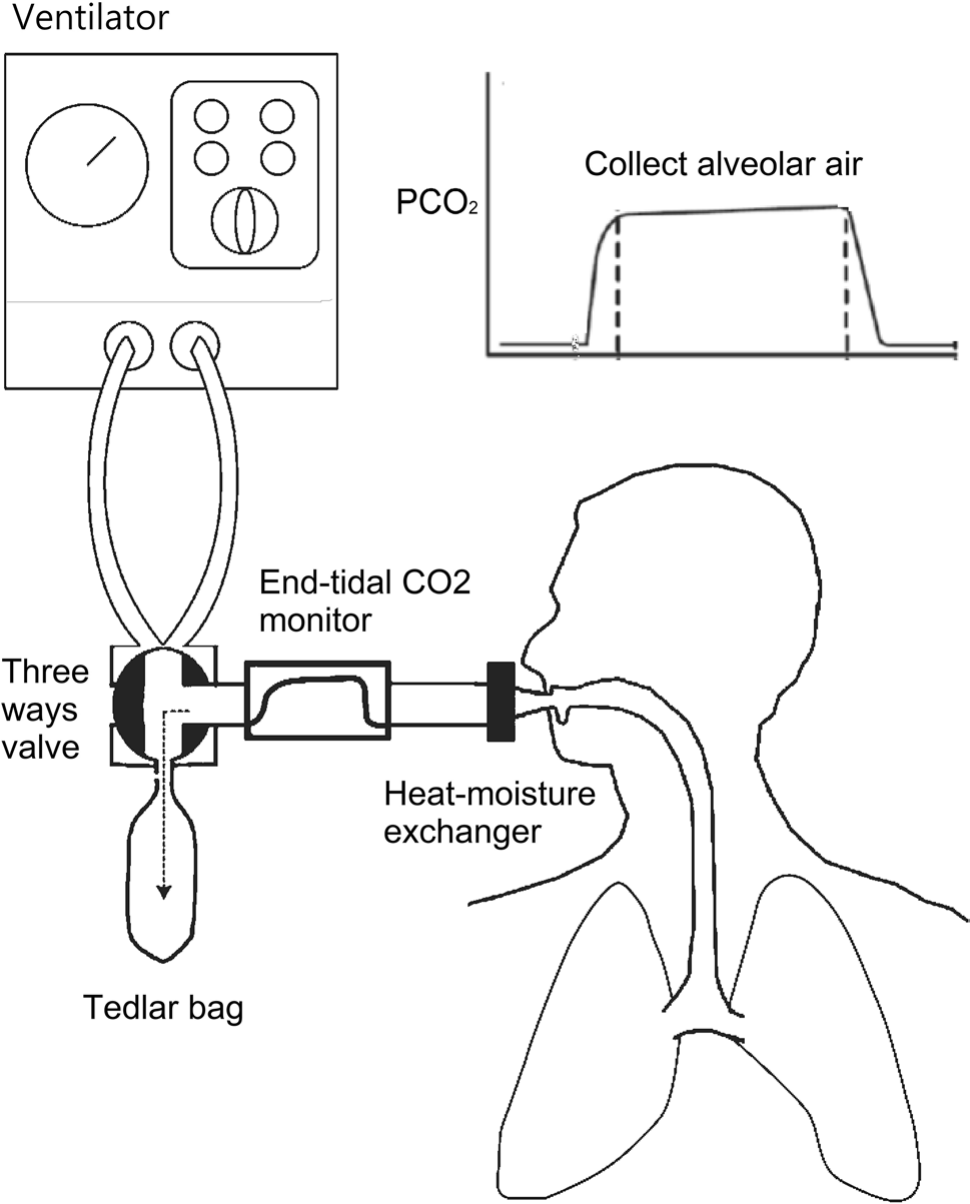
An alveolar air sampling by applying mainstream carbon dioxide monitoring and heat-moisture exchanger to remove dead space air and humidity of exhaled breath.

The strength of this study is that we sampled alveolar air directly from the tracheal tube to prevent contamination from the respiratory dead space, upper airway, and gastroenteric tract. The inclusion of dead space air in a breath sample may lead to variable dilution of breath sample and contamination from exogenous volatile organic compounds^18^. All subjects refrained from eating for at least eight hours before sampling and then underwent endotracheal intubation for surgery. This design can largely prevent contamination from the food odors in the gastroenteric tract and the oral cavity. We used a mainstream carbon dioxide monitor to guide the sampling of alveolar air. The anesthesiologist collected air only when the concentration of CO_2_ reached the highest level to ensure that the air came from the alveolar space. Compared with other studies, our sampling procedure can obtain the purest alveolar air with the highest concentration of volatile metabolites. Because humidity and temperature may have an influence on the electrical conductivity of the sensors and affect the measurement^19^, we connected a heat-moisture exchanger to keep a constant humidity and temperature (Fig. 5)^20^. Cigarette smoking affects volatile organic compounds in exhaled breath^21^. The study excluded subjects with a history of smoking or second-hand smoke. The purpose of strict exclusion criteria was to prevent the influence of smoking and other diseases and to provide the most reliable assessment of the breath test for breast cancer.

AI has gradually been used in the treatment decision support for breast cancer among oncologists with varying expertise^22^. Ha et al. developed a convolutional neural network algorithm to predict the molecular subtype of a breast cancer based on MRI features, and the test set accuracy was 70%, and the ROC was 0.853^23^. Park et al. conducted a radio-genomics study that investigated the accuracy of combing low-dose perfusion computed tomography and five machine learning models to predict molecular subtypes of invasive breast cancer, and results showed that the use of the random forest model had the best accuracy (66%) and AUC (0.82) to predict molecular subtype^24^. In the application of machine learning techniques in human studies, imbalance in class distribution may influence the performance of a classifier, and the random forest algorithm is suitable for class imbalance problems. Guo et al. compared the performance of four commonly used machine learning algorithms in high-dimensional omics data. They showed that the random forest was the best method when class distributions were unbalanced^25^. For sensor array data with imbalanced class distribution, Tan et al. reported that the random forest combined with the oversampling is an effective solution to improve the performance of the prediction model^26^. In this study, we also observed that the application of the random forest model had the highest accuracy to predict the molecular subtype of breast cancer.

To develop a new diagnostic test, it is important to assess not only the accuracy but also the reproducibility of results. Phillips et al. analyzed volatile organic compounds (VOCs) in the breath to diagnose breast cancer by GC–MS. At that study, five breath biomarkers (2-propanol, 2,3-dihydro-1-phenyl-4(1H)-quinazolinone, 1-phenyl-ethanone, heptanal, and isopropyl myristate) were identified and used to establish a prediction model that showed high accuracy^27^. Peng et al. conducted a similar study to explore the breath biomarkers (3,3-dimethyl pentane, 2-amino-5-isopropyl-8-methyl-1-azulenecarbonitrile, 5-(2-methylpropyl)nonane, 2,3,4-trimethyl, 6-ethyl-3-octyl ester 2-trifluoromethyl benzoic acid) of breast cancer by GC–MS^28^; however, the identified biomarkers were inconsistent with Phillips’s results^27^. Possible explanations for the discrepancy may include the effectiveness of VOC filters in preventing environmental contamination, subjective selection of candidate biomarkers, and the time interval between sampling and analysis that might change the composition or concentration of VOCs^27,28^. In this study, we applied alveolar air sampling and collected air from the lower respiratory tract to prevent any contamination from dead space or gastrointestinal tract, and all samples were analyzed immediately within 30 min. We have established standardized methods for the breath test, and all the procedures followed the STARD guideline to report a diagnostic accuracy study^29^. We have conducted a systemic review. We selected related studies published before November 20th, 2020, by searching PubMed and Web of Science. All relevant articles were retrieved without language or geographic limitations. The search terms breast cancer, breast tumor, sensor, and electronic nose were used in combination with the Boolean operators AND and OR. Studies were included if they met the following criteria: (1) observational studies: cross-sectional, case–control, or prospective designs; (2) population: breast cancer patients diagnosed according to the pathological report and established diagnostic systems; (3) studies that provided sufficient information of sensitivity, specificity, and accuracy; (4) studies that use an electronic nose to analyze endogenous VOC in feces, blood, exhaled breath, or urine to screen or assess breast cancer. The exclusion criteria were (1) duplicate publications; (2) letters or review articles; (3) cell or animal studies; (4) non-gas sensor. Our databases retrieved 699 articles. We excluded 652 articles by screening through the titles and abstracts. After a full-text review, we excluded a further 650, leaving two studies for inclusion^16,30^. Full details of the search results are provided in Supplementary Table S1. Because some confounding factors and comorbidities will affect diagnostic accuracy, and different studies used different exclusion criteria. We suggest that future studies could conduct a sensitivity analysis to show the impact of exclusion criteria and provide readers with an overall estimate of diagnostic accuracy.

The advantage of the electronic nose system is that it can perform rapid breath biopsy during the operation. We collected the alveolar air from the laryngeal mask airway and storage in a Tedlar air sampling bag and analyzed the sampled air offline in a room next to the operation room. We collected the air before surgery within a few minutes, and the analysis can be completed within 30 min during the surgery. Traditionally, it takes a week to get pathological and molecular studies reports.

However, there are some limitations. In this study, all subjects received anesthetics for surgery. Saraoglu et al. used quartz crystal microbalance E-nose sensors to predict the anesthetic dose level, and results showed that the anesthetics could be detected by the electronic nose^31^. In this study, we administered all study subjects with the anesthetic drug 2% Sevoflurane. We conservatively thought that the exhaled volatile organic compounds that distinguished the case group and the control group are not derived from the anesthetics. We recommend that future studies should also consider the possible effects of drugs during surgery. The intraoperative result obtained in this study cannot be directly applied outside the operating room.

## Conclusions

Cancer causes metabolic alteration to sustain fast cell growth and proliferation. The estrogen, progesterone, and human epidermal growth factor receptor 2 hormone receptors have a unique metabolomic expression in breast cancer patients. Analysis of the volatile metabolites in the breath of patients can be used to develop a breath test for breast cancer. This study used sensor array and machine learning algorithms to analyze breath samples from breast cancer patients. The results showed high accuracy and reliability in the discrimination of breast cancer and the molecular subtype. The novel breath test has great potential to develop a rapid breast cancer diagnostic tool during surgery.

## Methods

### Participants

We designed a case–control study to recruit cases of breast cancer and non-cancer controls. We consecutively recruited breast tumor patients who underwent breast tumor resection at the National Taiwan University Hospital. During the same period, we recruited a control group of subjects who underwent surgery for gall bladder stone, hernia, fractures, urinary incontinence, and uterine prolapse at the same hospital. The exclusion criteria included male gender, the history of asthma^14^, diabetes mellitus^14^, cigarette smoking^21^, receiving chemotherapy that may affect metabolism and influence volatile organic compounds in exhaled breath. We obtained medical history, occupational history, smoking history, medications, and dietary habits through face-to-face interviews and medical records. All subjects received blood tests of white blood cells, fasting sugar, blood urea nitrogen, creatinine, and alanine aminotransferase after eight hours of fasting.

All methods were carried out following relevant guidelines and regulations. The ethics committee of the National Taiwan University Hospital approved the research protocol (No. 201512102RINC). All subjects provided written informed consent before the study.

### Molecular subtype

This study used immunohistochemistry (IHC) to determine the status of ER, PR, and HER2. IHC was performed on formalin-fixed, paraffin-embedded tissue sections (thickness 4 μm) in the Central Pathology Laboratory at the hospital. ER and PR were determined using the Ventana Benchmark system (Ventana Medical Systems)^32^. The percentage of positive-staining nuclei was recorded. In this study, we applied the National Comprehensive Cancer Network (NCCN) criteria to determine breast cancer's molecular phenotype. Both ER and PR status were determined for all invasive breast cancer and ductal carcinoma in situ (DCIS) using a cutoff value of ≥ 1% as a positive result^33^. HER2 status was reported as strong positive when the IHC score was 3 +^34^. We defined the molecular subtype of breast cancer as (1) luminal A (ER-positive and/or PR-positive, and HER2-negative), (2) luminal B (ER-positive and/or PR-positive, and HER2-positive), (3) HER2/neu (ER-negative, PR-negative, and HER2-positive), and triple-negative (ER-negative, PR-negative, and HER2-negative).

### Collection of the breath sample

To avoid contamination from the dead space, we collected alveolar air sampling by applying mainstream carbon dioxide (CO_2_) monitoring^35^. All study subjects received a fixed dose of intravenous drugs for anesthetic induction. Sevoflurane 2% was administered after insertion of the laryngeal mask airway initially. The exhaled gas sampling was then performed. A heat-moisture exchanger was connected to the airway instrument to remove the humidity of exhaled breath. The anesthesiologist collected one-litter of alveolar air under the monitoring of the mainstream end-tidal CO_2_ analyzer before surgery. When the end-tidal CO_2_ concentration reached the plateau, the anesthesiologist opened the entrance of the three-way valve to sample the alveolar air into a Tedlar bag (Fig. 5).

### Analysis of E-nose

The collected air was analyzed using Cyranose 320 E-nose (Sensigent, California, USA) within 30 min, according to the established method^36^. The E-nose consists of 32 carbon nanotubes sensors that can measure the volatile organic compounds in the breath by the changes in sensor resistance^37^ (Supplementary Fig. 1). We analyzed all samples in the same room with a temperature of 19.5–23.9℃ and a humidity of 53–64%. The E-nose analyzed the air sample in each Tedlar bag ten times. According to the manufacturer’s suggestion and previous studies^36^, we eliminated the first measurement data and obtained the mean of the remaining measurements. The mean intra-class correlation coefficient (ICC) of sensor responses was 0.99 (SD 0.22) (Supplementary Table S2).

### Reference standard

This study confirmed the diagnosis of breast cancer based on pathology and immunohistochemistry reports. Using pathology and immunohistochemistry reports as the golden standard, we evaluated the validity and reliability of the breath test.

### Statistics

This study used eight machine learning algorithms to build prediction models, including *k*-nearest neighbors, naive Bayes, decision tree, neural network, support vector machines (SVMs) (including the linear kernel, polynomial kernel, and radial basis kernel), and random forest^38^. We randomly divided the data into a training set (80% of data) for model derivation and a test set (20% of data) for validation. We used the modelLookup function of the R caret package for automated parameter tuning to improve model performance^39^. We used a bootstrap method and calculated the accuracy of 100 iterations to decide the parameters of machine learning methods that had the highest prediction accuracy. Then, the optimized models were further tested in the independent test set to evaluate the accuracy. To prevent the influence of an unequal proportion of cases in each group, we adopted an oversampling method that replicates the observations of the minority class to balance the data^40^. We used the R package “class” to build the *k*-nearest neighbors model, “klaR” to build the naive Bayes model, “C50” to build the decision tree model, “neuralnet” to build the neural network model, “kernlab” to build the SVMs model, and “randomForest” to build the random forest model. We determined the validity of the breath test by accuracy, sensitivity, specificity, PPV, NPV, and AUC. AUC values of 0.7–0.8, 0.8–0.9, and 0.9–1.0 are regarded as good, very good, and excellent diagnostic accuracy, respectively^41^. To adjust accuracy by accounting for the possibility of a correct prediction by chance only, we also calculated an AUC with 2000 bootstrap replicates and the pAUC to assess the variability of the measure. The formula of pAUC was:

$$ pROC = \frac{1}{2}\left( {1 + \frac{pAUC - \min }{{\max - \min }}} \right) $$

where *min* is the pAUC over the same region of the diagonal ROC curve, and *max* is the pAUC over the same region of the perfect ROC curve^42^. Because we were interested in a diagnostic test with a high specificity and sensitivity, we also examined the partial AUC between 90 and 100% for specificity and sensitivity. We assessed the reliability by leave-one-out cross-validation and the kappa statistic. Kappa expresses the extent to which the observed agreement exceeds that would be expected by chance alone^43^. A kappa greater than 0.75 represents excellent agreement beyond chance, a kappa below 0.40 represents a poor agreement, and a kappa of 0.40 to 0.75 represents intermediate to good agreement.

To evaluate the influence of comorbidities and confounding factors on diagnostic accuracy, we conducted additional analyses to compare the effects of comorbidities and confounding factors on diagnostic accuracy. We included each potential confounding factor or comorbidity, used eight machine learning algorithms, and applied meta-analyses of diagnostic accuracy to generate pooled point estimates of the accuracy and SROC^44^. We used the DOR to quantify the impact of confounding factors on accuracy:

1 $$ {\text{DOR }} = \, \left( {{\text{True}}\,{\text{ positive}}/{\text{False }}\,{\text{negative}}} \right)/\left( {{\text{False }}\,{\text{positive}}/{\text{True }}\,{\text{negative}}} \right) $$

A DOR value ranges from 0 to infinity, with higher values indicating better discriminatory test performance. A value of 1 means that a test does not discriminate between patients with the disorder and those without it^45^. A test with a DOR of 10 is considered to be an excellent test^46^. Also, we included all subjects and did not exclude any confounding factor or comorbidity for readers to judge the worst-case scenario accuracy. The software used for this analysis was R-package mada.

## Supplementary Information

Supplementary Information.

Supplementary Table S1.

Supplementary Table S2.

Supplementary Figure S1.

## Data Availability

De-identified volatilome data is available upon request to the corresponding author.
