# Supplementary material for: Breath biopsy of breast cancer using sensor array signals and machine learning analysis
Source: Sci Rep. 2021 Jan 8;11:103. doi: 10.1038/s41598-020-80570-0 (PMC7794369; doi:10.1038/s41598-020-80570-0)
Supplement: Supplementary file 1 — Supplementary Information. [file 41598_2020_80570_MOESM1_ESM.pdf]

Title: Breath biopsy of breast cancer using sensor array signals and machine learning analysis

Authors: Hsiao-Yu Yang,[a,b] Yi-Chia Wang,[c,d], Hsin-Yi Peng,[a], and Chi-Hsiang Huang[c,d*]

Affiliations:
[a] Institute of Environmental and Occupational Health Sciences, National Taiwan University College of Public Health, Taipei, Taiwan
[b] Department of Environmental and Occupational Medicine, National Taiwan University Hospital, Taipei, Taiwan
[c] Department of Anesthesiology, National Taiwan University College of Medicine, Taipei, Taiwan
[d] Department of Anesthesiology, National Taiwan University Hospital, Taipei, Taiwan

Name and contact information for the corresponding author:
Chi-Hsiang Huang, M. D.
Assistant Professor
Department of Anesthesiology, National Taiwan University College of Medicine, Taipei, Taiwan
Department of Anesthesiology, National Taiwan University Hospital, Taipei, Taiwan
No.1 Jen Ai road section 1 Taipei 100 Taiwan
Tel.: 886-2-23562158
E-mail: tee.ntuh@gmail.com

Highlight:

- Cancer causes metabolic alteration to sustain fast cell growth and proliferation. The estrogen, progesterone, and human epidermal growth factor receptor 2 hormone receptors have a unique metabolomic expression in breast cancer patients.
- Analysis of the volatile metabolites in the breath of patients can be used to develop a breath test for breast cancer. This study used sensor array and machine learning algorithms to analyze breath samples from breast cancer patients.
- The results showed high accuracy and reliability in the discrimination of breast cancer and the molecular subtype. The novel breath test has great potential to develop a rapid breast cancer diagnostic tool during surgery.
