# Supplementary material for: Breath biopsy of breast cancer using sensor array signals and machine learning analysis
Source: Sci Rep. 2021 Jan 8;11:103. doi: 10.1038/s41598-020-80570-0 (PMC7794369; doi:10.1038/s41598-020-80570-0)
Supplement: Supplementary file 3 — Supplementary Table S2. [file 41598_2020_80570_MOESM3_ESM.pdf]

Title: Breath biopsy of breast cancer using sensor array signals and machine learning analysis

Authors: Hsiao-Yu Yang,[a,b] Yi-Chia Wang,[c,d], Hsin-Yi Peng,[a], and Chi-Hsiang Huang[c,d*]

Affiliations:

[a] Institute of Environmental and Occupational Health Sciences, National Taiwan University College of Public Health, Taipei, Taiwan

[b] Department of Environmental and Occupational Medicine, National Taiwan University Hospital, Taipei, Taiwan

[c] Department of Anesthesiology, National Taiwan University College of Medicine, Taipei, Taiwan

[d] Department of Anesthesiology, National Taiwan University Hospital, Taipei, Taiwan

Name and contact information for the corresponding author:

Chi-Hsiang Huang, M. D.
Assistant Professor
Department of Anesthesiology, National Taiwan University College of Medicine, Taipei, Taiwan
Department of Anesthesiology, National Taiwan University Hospital, Taipei, Taiwan
No.1 Jen Ai road section 1 Taipei 100 Taiwan
Tel.: 886-2-23562158
E-mail: tee.ntuh@gmail.com

Supplementary Table S1. Studies on the electronic nose in breast cancer

Author	Type of sensor	Number of patients	Number of controls	Cancer Stage	VOC sources	Sensitivity	Specificity	AUC	Exclusion criteria
Barash et al., [1] 2015	Gold nanoparticles, single-wall carbon nanotubes	169	82	N/A	Breath	84% (test set)	80% (test set)	0.90 (test set)	(1) chronic diseases, such as diabetes, coronary heart disease, renal insufficiency, rheumatoid; (2) local or systemic infection; (3) with obvious obstruction in the lung ventilation; (4) received chemotherapy, surgery, or interventional therapy; (5) history of other tumors
Diaz de Leon-Martinez, [2] 2020	Carbon nanotube-polymer	262	181	0-IV	Breath	100%	100%	0.98	(1) without fasting conditions, (2) without smoking, (3) without oral hygiene, (4) taking any medication

Reference

1 Moses, L. E., Shapiro, D. & Littenberg, B. Combining Independent Studies of a Diagnostic-Test into a Summary Roc Curve - Data-Analytic Approaches and Some Additional Considerations. *Stat. Med.* **12**, 1293-1316, doi:DOI 10.1002/sim.4780121403 (1993).

2 Diaz de Leon-Martinez, L. *et al.* Identification of profiles of volatile organic compounds in exhaled breath by means of an electronic nose as a proposal for a screening method for breast cancer: a case-control study. *J Breath Res* **14**, 046009, doi:10.1088/1752-7163/aba83f (2020).
