# Supplementary material for: Breath biopsy of breast cancer using sensor array signals and machine learning analysis
Source: Sci Rep. 2021 Jan 8;11:103. doi: 10.1038/s41598-020-80570-0 (PMC7794369; doi:10.1038/s41598-020-80570-0)
Supplement: Supplementary file 4 — Supplementary Figure S1. [file 41598_2020_80570_MOESM4_ESM.pdf]

Title: Breath biopsy of breast cancer using sensor array signals and machine learning analysis

Authors: Hsiao-Yu Yang,[a,b] Yi-Chia Wang,[c,d], Hsin-Yi Peng,[a], and Chi-Hsiang Huang[c,d*]

Affiliations:

[a] Institute of Environmental and Occupational Health Sciences, National Taiwan University College of Public Health, Taipei, Taiwan

[b] Department of Environmental and Occupational Medicine, National Taiwan University Hospital, Taipei, Taiwan

[c] Department of Anesthesiology, National Taiwan University College of Medicine, Taipei, Taiwan

[d] Department of Anesthesiology, National Taiwan University Hospital, Taipei, Taiwan

Name and contact information for the corresponding author:

Chi-Hsiang Huang, M. D.
Assistant Professor
Department of Anesthesiology, National Taiwan University College of Medicine, Taipei, Taiwan
Department of Anesthesiology, National Taiwan University Hospital, Taipei, Taiwan
No.1 Jen Ai road section 1 Taipei 100 Taiwan
Tel.: 886-2-23562158
E-mail: tee.ntuh@gmail.com

Supplementary Table S2. The intra-class correlation coefficients (ICCs) of sensor responses.

	Measurement							
	**3rd**	**4th**	**5th**	**6th**	**7th**	**8th**	**9th**	**10th**
**Mean**	0.998	0.998	0.997	0.996	0.992	0.993	0.993	0.992
**SE**	0.001	0.000	0.001	0.001	0.001	0.001	0.001	0.001

SE: standard error.
